# Trends in diagnostic prevalence and treatment patterns of male and female ankylosing spondylitis patients in the United States, 2006–2016

**DOI:** 10.1186/s41927-019-0086-3

**Published:** 2019-09-23

**Authors:** Jessica Walsh, Theresa Hunter, Krista Schroeder, David Sandoval, Rebecca Bolce

**Affiliations:** 1 University of Utah, Salt Lake City, UT USA; 2 Eli Lilly and Company, Indianapolis, Indiana USA

**Keywords:** Ankylosing spondylitis, Prevalence, Treatment patterns

## Abstract

**Background:**

There has been much variation between epidemiological studies that report the prevalence of ankylosing spondylitis (AS). This study aimed to analyze the diagnostic prevalence rates and treatment patterns of male and female AS patients in the United States adult insured population from 2006 to 2016.

**Methods:**

Trends in AS prevalence were calculated for the 11-year period covering January 1, 2006 to December 31, 2016. Adult (18+ years old) AS patients were included in this retrospective analysis of medical and pharmacy claims data from the IBM Marketscan Commercial, Medicaid and Medicare-Supplemental Claims database. Prevalence was determined as having ≥1 AS diagnostic codes (ICD-9:720.0; ICD-10:M45.x). Trends in treatment patterns were also analyzed and stratified by gender.

**Results:**

The AS prevalence increased from 0.04 to 0.09% from 2006 to 2016. The mean age between 2006 and 2016 ranged from 49.52–50.00 years. In 2006, approximately 40% of AS patients were female, while in 2016 over 47% of AS patients were female. Rates of use of TNF inhibitors and oral glucocorticoids increased, while NSAIDs and non-biologic DMARDs (sulfasalazine & methotrexate) rates decreased. Opioid use rates were stable. In 2016, males were more likely to be prescribed biologics, while females were more likely to be prescribed methotrexate, sulfasalazine, NSAIDs, muscle relaxants, anticonvulsants, opioids, and glucocorticoids.

**Conclusions:**

The prevalence of AS diagnosis codes more than doubled between 2006 and 2016, but the very low prevalence suggests that AS continues to be underdiagnosed and under-addressed in routine clinical practice. Despite the increase in female AS patients, females were less likely to be prescribed biologics compared to male AS patients.

## Background

Ankylosing spondylitis (AS) is a chronic inflammatory disease that affects the spine and sacroiliac joints [1]. AS is associated with pain, impaired health-related quality of life (HRQoL) and disability [2, 3]. Musculoskeletal features as well as extra-articular manifestations such as enthesitis, uveitis, inflammatory bowel disease, and psoriasis can cause a substantial burden to AS patients. There has been much variation between epidemiological studies that report the prevalence of AS [4–6]. Given these discrepancies, the prevalence of AS in the United States (US) has been estimated to be between 0.2 and 1% [7–9].

Treatment options for AS patients include nonsteroidal anti-inflammatory drugs (NSAIDs), nonbiological disease-modifying antirheumatic drugs (DMARDs) such as sulfasalazine and methotrexate, and biologics such as tumor necrosis factor inhibitors (TNFi) and interleukin-17 (IL-17) antagonists [10–16]. The American College of Rheumatology (ACR), Spondylitis Association of America (SAA), and Spondyloarthritis Research and Treatment Network (SPARTAN) recommend NSAIDs as a first-line treatment for AS and biologic therapy for patients that do not respond to NSAIDs [16].

The purpose of this study was to assess the current diagnostic prevalence of AS and trends in treatment patterns among adults in the US that have commercial, Medicaid, or Medicare supplemental insurance. In order to do this, data from administrative insurance claims databases over the period 2006 to 2016 were analyzed.

## Methods

### Study design

This retrospective cross-sectional study utilized data from the IBM MarketScan® Research database (Ann Arbor, MI, USA). Data were analyzed to assess trends in AS diagnostic prevalence focusing on the 11-year period from January 1, 2006 to December 31, 2016. AS diagnostic prevalence rates were analyzed for the total AS population and stratified by age and gender. Demographic variables and patient characteristics were assessed and the age-adjusted prevalence rate was measured and stratified for 2016 cohort.

IBM Marketscan Commercial, Medicaid and Medicare-Supplemental Claims database contains de-identified patient data including in-patient and outpatient physician visits, emergency room visits, procedures, and pharmacy prescriptions. Study variables were defined in the IBM Marketscan database using patient enrollment records and International Classification of Diseases, 9th Revision, Clinical Modification (ICD-9-CM) and International Classification of Disease, 10th Revisions, Clinical Modification (ICD-10-CM) codes. In order to protect patient privacy, all data from the IBM Marketscan Research database are compliant to the Health Insurance Portability and Accountability Act (HIPAA).

### Identification of AS

For each calendar year of analysis, a cohort was assembled that consisted of all AS patients that were 18 years or older on January 1st of the calendar year. Patients were required to have continuous enrollment in medical and pharmacy benefits throughout the calendar year, with the exception of an enrollment gap allowance of less than 30 days. Patients with at least one AS diagnostic code (ICD-9:720.0; ICD-10: M45.x) were identified from these cohorts.

### Prevalence estimation

Annual AS diagnostic prevalence was estimated using the US adult population in the IBM MarketScan® Research database during the 11-year period of 2006 to 2016. For each calendar year, a cohort was created and the AS case identification was applied. The numerator in the AS diagnostic prevalence estimation was the number of patients that met the AS definition described in the previous section. The denominator was the number of all patients over the age of 18 with continuous enrollment (during the calendar year) in the cohort.

### Statistical analyses

AS diagnostic prevalence was estimated and stratified by gender (male and female) and age (< 25, 25–34, 35–44, 45–54, 55–64, and ≥ 65) for each calendar year from 2006 to 2016. Trends in treatments patterns of tumor necrosis factor inhibitors (TNFi) (adalimumab, infliximab, etanercept, golimumab, certolizumab pegol), secukinumab, Cox-2 inhibitor (celecoxib), sulfasalazine, methotrexate, acetaminophen, NSAIDs (aspirin, ibuprofen, meloxicam, nabumetone, diclofenac, naproxen, diflunisal, etodolac, fenoprofen, fluribiprofen, indomethacin, ketoprofen, ketorolac, meclofenamate, mefanamic, meprobamate, oxaprozin, piroxicam, sulindac, tolmetin, salsalate), muscle relaxants (cyclobenzaprine, orphenadrine, chlorzoxazone, methocarbamol, carisoprodol, metaxalone, dantrolene, baclofen, tizanidine), anticonvulsant (gabapentin, pregabalin, carbamazepine, topiramate, oxcarbazepine), opioids (codeine, oxycodone, hydrocodone, propoxyphene, dihydrocodeine, fentanyl, hydromorphone, levorphanol, methadone, morphine, oxymorphone, tramadol, tapentadol, meperidine, butorphanol, buprenorphine, nalbuphine, pentazocine), and oral and injectable glucocorticoids (betamethasone, cortisone, dexamethasone, hydrocortisone, methylprednisolone, prednisolone, prednisone, triamcinolone). Descriptive statistics of patient demographics and treatment patterns were conducted for the total AS cohort and male and female subgroups. Continuous variables were analyzed by means and standard deviations (SD) while categorical variables were analyzed by frequency counts and percentages (%).

## Results

### AS patient characteristics in 2016

**Fig. 1:**
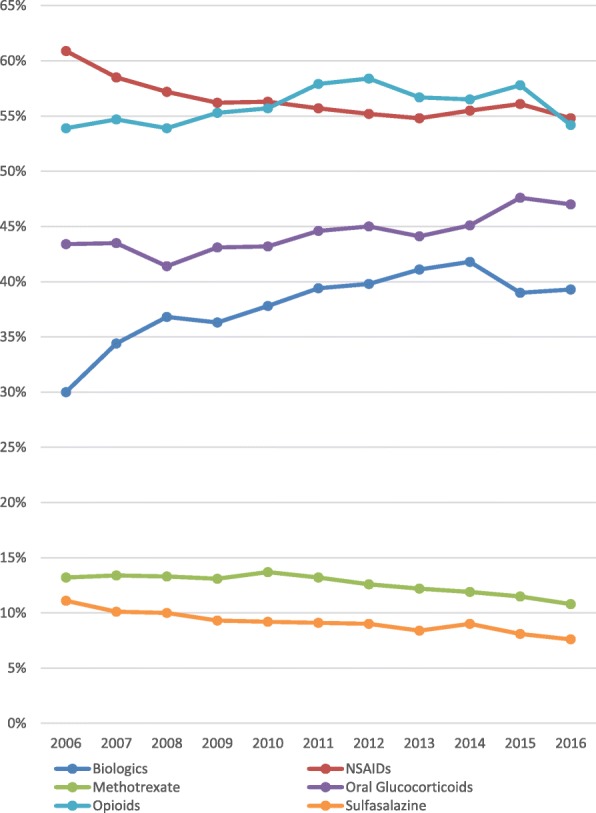
Medication use among male and female as patients in 2016

In 2016, out of a total of 16,097,378 adult patients that had commercial insurance, Medicaid, or supplemental Medicare with continuous enrollment in the IBM MarketScan® Research database, there were 14,729 (0.09%) patients with AS. Of these 14,729 patients, 7842 (53.24%) were male and 6887 (46.76%) were female. Mean age for overall AS population was 50.00 years (SD = 14.36). A majority of patients were commercially insured and the most frequent geographic location was the Southern US region. In 2016, males were more likely to be prescribed biologics, while females were more likely to be prescribed methotrexate, sulfasalazine, NSAIDs, muscle relaxants, anticonvulsants, opioids, and glucocorticoids (Fig. 1). The patients’ demographic information is presented in Table 1.

**Table 1:** Baseline characteristics for patients with ankylosing spondylitis (2016)

Variable	Overall Population (*N* = 14,729)	Males (*N* = 7842)	Females (*N* = 6887)
Mean Age (SD)	50.00 (14.36)	50.69 (14.62)	49.21 (14.02)
Age (Years)			
< 25	672 (4.6%)	332 (4.2%)	340 (4.9%)
25–34	1530 (10.4%)	843 (10.7%)	687 (10.0%)
35–44	2930 (19.9%)	1482 (18.9%)	1448 (21.0%)
45–54	3806 (25.8%)	1883 (24.0%)	1923 (27.9%)
55–64	3909 (26.5%)	2160 (27.5%)	1749 (25.4%)
≥ 65	1882 (12.8%)	1142 (14.6%)	740 (10.7%)
Insurance			
Commercial	12,687 (86.1%)	6605 (84.2%)	6082 (88.3%)
Medicare	2149 (14.6%)	1307 (16.7%)	842 (12.2%)
Geographic Region			
Northeast	2423 (16.5%)	1348 (17.2%)	1075 (15.6%)
North Central	2953 (20.0%)	1693 (21.6%)	1260 (18.3%)
South	6400 (43.5%)	3244 (41.4%)	3156 (45.8%)
West	2895 (19.7%)	1528 (19.5%)	1367 (19.8%)
Unknown	58 (0.4%)	29 (0.4%)	29 (0.4%)
Medication			
Biologics	5795 (39.3%)	3384 (43.2%)	2411 (35.0%)
Cox-2 inhibitor	1154 (7.8%)	569 (7.3%)	585 (8.5%)
Sulfasalazine	1122 (7.6%)	528 (6.7%)	594 (8.6%)
Methotrexate	1591 (10.8%)	695 (8.9%)	896 (13.0%)
Acetaminophen	425 (2.9%)	118 (1.5%)	307 (4.5%)
NSAIDs	8065 (54.8%)	3970 (50.6%)	4095 (59.5%)
Muscle Relaxants	4273 (29.0%)	1823 (23.2%)	2450 (35.6%)
Anticonvulsants	2859 (19.4%)	1110 (14.2%)	1749 (25.4%)
Opioids	7980 (54.2%)	3897 (49.7%)	4083 (59.3%)
Oral Glucocorticoids	6928 (47.0%)	3251 (41.5%)	3677 (53.4%)
Injectable Glucocorticoids	4832 (32.8%)	2115 (27.0%)	2717 (39.5%)

### Ankylosing spondylitis prevalence and treatment patterns: 2006–2016

**Fig. 2:**
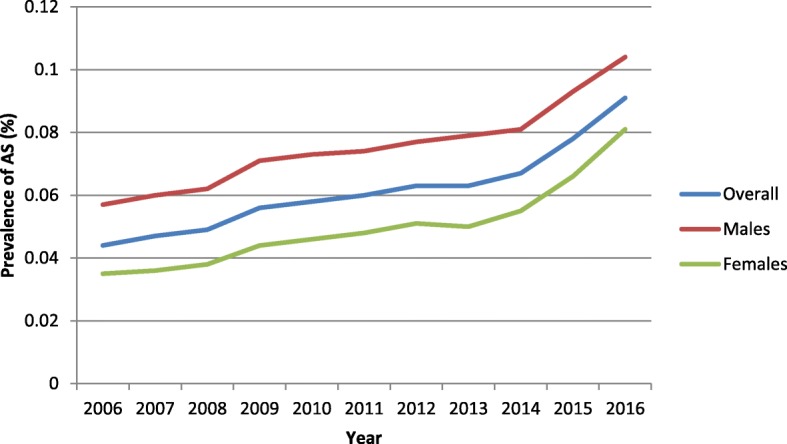
Ankylosing spondylitis prevalence trends stratified by gender (2006–2016)

Annual AS diagnostic prevalence rates increased from 0.04 to 0.09% from 2006 to 2016 for adult US patients in the IBM MarketScan® Research database. The diagnostic prevalence rate varied by age and gender during each year and increased gradually across the years for males and females. Overall diagnostic prevalence in males gradually increased from 0.06% in 2006 to 0.10% in 2016, while the overall diagnostic prevalence among females gradually increased from 0.03% in 2006 to 0.08% in 2016 (Fig. 2). In 2006, approximately 40% of AS patients were female, while in 2016 over 47% of AS patients were female. Mean age was stable across the years with an overall mean age of 49.52 in 2006 and 50.00 in 2016.

**Fig. 3:**
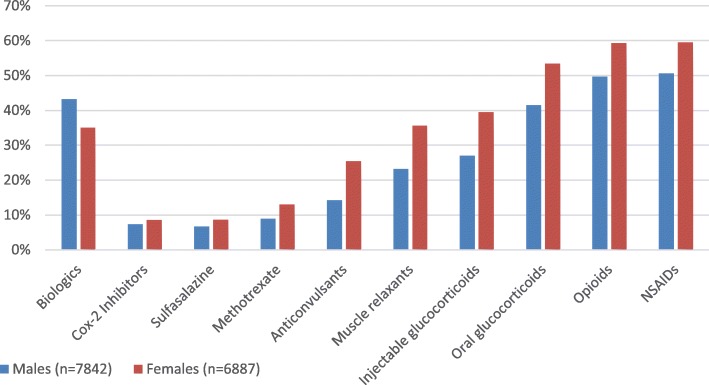
Trends in treatment patterns among as patients, 2006–2016

Rates of use of TNFi (30.00 to 39.30%) and oral glucocorticoids (43.40 to 47.00%) increased, while NSAIDs (60.90 to 54.80%) and non-biologic DMARDs (sulfasalazine (11.10 to 7.50%) & methotrexate (13.20 to 10.80%)) rates decreased from 2006 to 2016. Opioid use rates were stable from 2006 to 2016 (Fig. 3).

## Discussion

This study evaluated recent trends in diagnostic prevalence of AS and treatment patterns among AS patients in the U.S. Results from this study indicate that the overall diagnostic prevalence of AS in the U.S. ranged from 0.04 to 0.09% and steadily increased from 2006 to 2016 in a U.S. administrative claims database.

When analyzing 1996–2009 computerized health care data from Kaiser Permanente Northern California, Curtis and colleagues reported a point prevalence of AS standardized to the 2000 US census of 2.26 per 1000 (~ 0.23%) [8]. These findings were higher than the rates reported during our study, but lower than previously reported prevalence rates in the U.S. For example, based on the 2009–2010 NHANES data, axSpA (AS and nr-axSpA) prevalence was approximately 1.4% in the U.S. [7]. In addition Strand and colleagues [9] estimated that AS affects nearly 0.3% of the U.S. population ages 18–44 years in a retrospective chart review of at-risk patients in U.S. rheumatology practice. While the Reveille [7] and Strand [9] studies have been frequently referenced, it is important to note that these studies were not calculating diagnostic code prevalence using an administrative claims database. Differences in patient identification, data collection methods, and study design may contribute to the variability in results between studies. The inclusion of previously undiagnosed patients and nr-axSpA patients contributed to the particularly higher prevalence in the Reveille study [7].

Our study found that AS trends in the U.S. have gradually increased from 2006 to 2016 in both males (0.06 to 0.10%) and females (0.03 to 0.08%). Although males had greater prevalence of AS throughout the study, females went from accounting for 40% of the total AS population in 2006 to accounting for 47% of the total AS population in 2016. These findings are similar to the results reported in Ontario, Canada which saw a twofold increase among males (0.10 to 0.24%) and a threefold increase among females (0.06 to 0.19%) from 1995 to 2010 [17]. Increased awareness of AS and changing perceptions about male predominance of AS may have contributed to the increase in rates of diagnostic prevalence.

Rates of use of TNFi and oral glucocorticoids increased, while NSAIDs and non-biologic DMARDs (sulfasalazine & methotrexate) rates decreased from 2006 to 2016. Over the past decade, there has been an increase in TNFi options and a plethora of data on the safety and efficacy of TNFi, which has resulted in physicians becoming more comfortable with prescribing these medications [10–16]. The ACR, SAA, and SPARTAN recommend against the use of non-biologic DMARDs in most patients with AS, that has remained active despite NSAID use, and recommends the use of TNFi; which could also contribute to these changes in treatment patterns [16]. While NSAIDs can alleviate symptoms, the decreasing rate of NSAID use may be attributed to the associated risks with long-term administration and the potential cardiovascular, gastrointestinal, and renal complications [16].

Despite the increase in TNFi use among AS patients, there were still different rates of use among males and females. In 2016, males were more likely than females to be prescribed TNFi. These findings may indicate that female patients with AS are not being prescribed advanced therapy at the same rate as their male counterparts. This may be due to prescribers being more reluctant to treat women with “more aggressive” therapy since there are still misconceptions that AS is a male dominated disease however reasons for not receiving TNFi were not analyzed in this study.

While this study provides insight into the diagnostic prevalence rate and trends in treatment patterns of AS patients in the United States, it is important to note that the primary purpose of insurance claims data is for administrative and billing purposes. Since our study analyzed the diagnostic prevalence of AS patients by identifying patients with ICD-9 and/or ICD-10 codes, our findings could be an under-representation of the overall AS population. As reported by Curtis et al., using different case definitions based on the same ICD codes may lead to variation in the prevalence estimates [8]. Published reports have indicated a delay in diagnosis of up to 10 years for AS patients [18, 19]. which means that many patients with AS may not currently be diagnosed. Diagnostic prevalence rates would not include these patients in the calculation and could thus be an underestimation of the total AS prevalence in the US. Since our analyses were based on ICD-9 and ICD-10 codes, we are not able to assess if these calculations include patients that may have nonradiographic axial spondyloarthritis, but received an AS diagnostic code..

Additionally, since the IBM MarketScan® Research database consists of patients with commercial, Medicaid, and/or Medicare supplement insurance, this may inadvertently exclude some populations from the analyses such as the uninsured population or patients with other insurances. Given the limitations of the database used in this study, all AS patients may not be included in our analyses, thus resulting in a conservative estimate of AS diagnostic prevalence in the U.S.

## Conclusion

The large sample size and geographic representation of our study enhances the validity of generalizing our AS diagnostic prevalence estimates to the general U.S. adult population that are insured by commercial insurance, Medicaid, or supplemental Medicare. The prevalence of AS diagnosis codes more than doubled between 2006 and 2016, but the very low prevalence suggests that AS continues to be underdiagnosed and under-addressed in routine clinical practice. Despite the increase in female AS patients, females were less likely to be prescribed biologics compared to male AS patients. The reasons why female patients were less likely to be prescribed biologics were not investigated in this study and additional research is needed to understand the difference in the treatment patterns between male and female AS patients.

## Data Availability

All data used in this study was de-identified. Request for data may be made to the corresponding author.

## Abbreviations

ACR: American College of Rheumatology
AS: Ankylosing Spondylitis
AxSpA: Axial Spondyloarthritis
DMARD: Disease-modifying antirheumatic drug
HIPPA: Health Insurance Portability and Accountability Act
HRQoL: Health related quality of life
ICD-10-CM: International Classification of Disease, 10th Revisions, Clinical Modification
ICD-9-CM: International Classification of Diseases, 9th Revision, Clinical Modification
NSAID: Nonsteroidal anti-inflammatory drug
SAA: Spondylitis Association of America
SD: Standard deviation
SPARTAN: Spondyloarthritis Research and Treatment Network
TNFi: Tumor necrosis factor inhibitor
U.S: United States
